# Stereotactic Body Radiotherapy (SBRT) for liver metastasis – clinical outcomes from the international multi-institutional RSSearch® Patient Registry

**DOI:** 10.1186/s13014-018-0969-2

**Published:** 2018-02-13

**Authors:** Anand Mahadevan, Oliver Blanck, Rachelle Lanciano, Anuj Peddada, Srinath Sundararaman, David D’Ambrosio, Sanjeev Sharma, David Perry, James Kolker, Joanne Davis

**Affiliations:** 1 Geisinger Health System, 100 N Academy Ave, Danville, PA 17822 USA; 2 Department of Radiation Oncology, University Medical Center Schleswig-Holstein, Kiel, Germany; 3 Saphir Radiosurgery Center Guestrow, Frankfurt, Germany; 4 Department of Radiation Oncology, Crozer Keystone Health Care Center, Philadelphia CyberKnife, Havertown, PA USA; 5 Department of Radiation Oncology, Centura Health, Colorado Springs, CO USA; 6 Department of Radiation Oncology, Memorial Regional Hospital, Hollywood, FL USA; 7 Department of Radiation Oncology, New Jersey CyberKnife at Community Medical Center, Toms River, NJ USA; 8 Department of Radiation Oncology, St. Mary’s Medical Center, Huntington, WV USA; 9 Department of Radiation Oncology, Medstar Franklin Square Medical Center, Baltimore, MD USA; 10 Department of Radiation Oncology, University of Pennsylvania, Philadelphia, PA USA; 11 The Radiosurgery Society, San Mateo, CA USA

**Keywords:** SBRT, Liver metastasis, RSSearch registry

## Abstract

**Background:**

Stereotactic body radiotherapy (SBRT) is an emerging treatment option for liver metastases in patients unsuitable for surgery. We investigated factors associated with clinical outcomes for liver metastases treated with SBRT from a multi-center, international patient registry.

**Methods:**

Patients with liver metastases treated with SBRT were identified in the RSSearch® Patient Registry. Patient, tumor and treatment characteristics associated with treatment outcomes were assessed. Dose fractionations were normalized to BED_10_. Overall survival (OS) and local control (LC) were evaluated using Kaplan Meier analysis and log-rank test.

**Results:**

The study included 427 patients with 568 liver metastases from 25 academic and community-based centers. Median age was 67 years (31–91 years). Colorectal adenocarcinoma (CRC) was the most common primary cancer. 73% of patients received prior chemotherapy. Median tumor volume was 40 cm^3^ (1.6–877 cm^3^), median SBRT dose was 45 Gy (12–60 Gy) delivered in a median of 3 fractions [1–5]. At a median follow-up of 14 months (1–91 months) the median overall survival (OS) was 22 months. Median OS was greater for patients with CRC (27 mo), breast (21 mo) and gynecological (25 mo) metastases compared to lung (10 mo), other gastro-intestinal (GI) (18 mo) and pancreatic (6 mo) primaries (*p* < 0.0001). Smaller tumor volumes (< 40 cm^3^) correlated with improved OS (25 months vs 15 months *p* = 0.0014). BED_10_ ≥ 100 Gy was also associated with improved OS (27 months vs 15 months p < 0.0001). Local control (LC) was evaluable in 430 liver metastases from 324 patients. Two-year LC rates was better for BED_10_ ≥ 100 Gy (77.2% vs 59.6%) and the median LC was better for tumors < 40 cm^3^ (52 vs 39 months). There was no difference in LC based on histology of the primary tumor.

**Conclusions:**

In a large, multi-institutional series of patients with liver metastasis treated with SBRT, reasonable LC and OS was observed. OS and LC depended on dose and tumor volume, while OS varied by primary tumor. Future prospective trials on the role of SBRT for liver metastasis from different primaries in the setting of multidisciplinary management including systemic therapy, is warranted.

**Trial registration:**

Clinicaltrials.gov: NCT01885299.

## Background

Metastatic lesions to the liver from other primary sites are not uncommon and can be a significant burden for patients, caregivers and health care providers. Liver metastases can cause significant morbidity with pain and anorexia, adversely affecting health-related quality of life. In addition, more extensive liver disease can cause hepatic dysfunction and worsening performance status limiting systemic therapy and increasing mortality [1]. The most common metastatic lesion in the liver is from colorectal adenocarcinoma [2]. In 2017, the incidence of colorectal cancer (CRC) in the United States is estimated to be approximately 135,430 new cases, with half of these patients going on to develop liver metastasis in their lifetime [2, 3]. Clinical series and autopsy studies have shown that as many as 40–50% of patients with metastatic CRC have disease confined to the liver [4], many oligometastatic [5], making these patients amenable for liver-directed therapies. Surgical hepatic metastatectomy has a long track record with 5-year survival rates of 50%–60% and up to 20% can achieve long-term disease-free survival in carefully selected patients [6–8]. However, only 10%–20% of liver metastases are amenable to resection, leaving systemic therapy as the traditional recourse for majority of patients. For unresectable tumors, despite advances in combination chemotherapy and targeted agents resulting in a doubling of median survival from approximately 10 to 20 months, it is not without significant toxicity [9]. Chemotherapy has also been used to downstage lesions, potentially allowing patients to become eligible for surgery [10]. Since most patients with liver metastases remain ineligible for surgery, alternative liver-directed therapies, such as stereotactic body radiotherapy (SBRT), radiofrequency ablation, microwave ablation, radiolabeled microspheres, transarterial chemo embolization, cryoablation, and alcohol injection, have shown some benefit [11].

Radiation therapy is an established palliative modality [12], and for patients experiencing painful liver metastasis, even a single fraction of external beam radiotherapy directed to the whole liver can achieve meaningful symptomatic relief and improved quality of life in a majority of patients [13]. However, for eradicating metastatic disease, conventional radiation therapy techniques treating large areas of the liver are largely ineffective owing to the low tolerance of the liver to high-dose irradiation due to the risk of radiation-induced liver disease (RILD) [14–16]. With the emergence of more sophisticated treatment planning software and methods of image guidance in the past two decades, more tightly focused treatment fields are now possible, allowing for delivery of higher doses in fewer fractions to discrete individual liver lesions, while sparing the uninvolved liver [17–21]. With this combination of spatial precision and the administration of tumoricidal radiation doses with SBRT, it is feasible to achieve high rates of tumor control while minimizing the irradiation of surrounding healthy tissue, thereby reducing the risk of RILD [22].

Multiple retrospective and prospective series have explored the feasibility, efficacy and safety of SBRT for liver metastasis [23–32]. The purpose of this study is to report on clinical outcomes and the tumor and treatment characteristics related to these outcomes, in patients with liver metastasis treated and enrolled in RSSearch® Patient Registry, an international database dedicated to collecting data from patients treated with radiosurgery and SBRT. RSSearch® currently includes over 20,000 patients treated with radiosurgery and SBRT and outcome analysis has been reported on large cohorts of patients, for example, in early-stage lung cancer or lung metastasis with over 700 lesions treated with SBRT [33–35].

## Methods

A retrospective analysis of patients with liver metastases and enrolled in the RSSearch® Patient Registry (Clinicaltrials.gov Identifier: NCT01885299) was performed. RSSearch® is managed by the Radiosurgery Society®, a non-profit professional medical society. A description of the methodology, database design and initial patient and treatment characteristics of patients enrolled in RSSearch® has been previously reported [36]. The database was housed by an independent third-party, Advertek^SM^, Inc. (Louisville, KY) and has been migrated and currently housed with VisionTree (San Diego, CA). The hosts meet all requirements to comply with the Health Insurance Portability and Accountability Act (HIPAA) and Safe Harbor Policy to maintain system security, transmission of data and patient confidentiality. All centers treating patients with SBRT clinically are offered and encouraged to participate in RSSearch®. Participation is voluntary and no compensation is provided either to patients or participating centers. Each principal investigator is provided a copy of the RSSearch® Registry protocol, case report forms, sample patient informed consent, and web-based training for data entry and database navigation. Local Institutional Review Board/Ethics Committee (IRB/EC) approval is required at all participating centers. Informed consent was obtained from all patients, as required by individual IRB/ECs, prior to the patient’s data entered the RSSearch® Registry. The selection of centers for this study included RSSearch® participating centers that treated patients with liver metastasis with SBRT between March 2005 to January 2017, with complete data entry fields for screening, treatment and follow-up (minimum survival data) for their respective patients. Patients were treated at 25 institutions located in the US, Germany and Australia.

As this was a registry, there were no protocol defined patient selection and treatment planning criteria and treatment was done per institutional guidelines based on existing literature. All patients had fiducial markers placed in and around the tumor, usually by interventional radiology. In general, all patients were simulated supine, in the treatment position. Planning computed tomography (CT) scans were obtained above and below the region of interest in expiration, inspiration and free breathing. One-millimeter slice thickness reconstructions in the axial plane were transferred to the treatment planning station. Target volumes were delineated by the treating radiation oncologist, with the help of the radiologist or surgeon when necessary, using all available imaging studies, typically including at least CT and often MRI scanning. The gross tumor volume (GTV) was generally used as the clinical target volume (CTV), and a margin of 3–10 mm was used to delineate the planning target volume (PTV). All planning was performed using inverse planning on the MultiPlan® System (Accuray Incorporated, Sunnyvale, CA) allowing non-isocentric, and non-coplanar radiation delivery using either a ray tracing algorithm or Monte Carlo calculations. All patients were treated using CyberKnife® Stereotactic Radiosurgery System (Accuray Incorporated, Sunnyvale, CA). Real-time tumor tracking was accomplished using Synchrony® Respiratory Motion Tracking System, which synchronizes the beam delivery with the motion of the target resulting from respiration, without the need to interrupt the treatment or move the patient. All patients were treated per the respective institutional guidelines. To compare the effects of various treatment protocols with different treatment fraction sizes and doses, the biologically effective dose (BED) was calculated using the linear quadratic model; as BED = D*(1 + d/α/β) where D is the total dose, d is the dose per fraction and the α/β ratio for the tumor was 10 Gy. Normal tissue dose restraints were reported by the treating institutions and captured in RSSearch® as the maximum point dose and interquartile range for each structure.

Patient follow-up was performed per institutional guidelines. All participating centers reported follow-up clinical and imaging data. Local control (LC) was evaluated independently for each lesion at the participating institution following a modified RECIST (Response Evaluation and Criteria in Solid Tumors) criteria. Local progression was defined as greater than 20% increase in the size of lesions and/or appearance of one or more lesions in target treatment location, LC defined as disappearance of, decrease in, or no increase in size of the treated lesions. An independent audit was conducted on 10% sample of patients to assess accuracy and completeness of RSSearch® data. Any missing information or deviations were immediately reported to the participating center and corrections were made prior to data analysis.

Analyses of LC, and OS were calculated using the Kaplan-Meier method. LC was analyzed for each treated tumor whereas analysis of OS was calculated for every patient. Specific cause of death was not reported for all patients in RSSearch® and therefore not evaluated in this study. Subgroups were compared using X^2^ and log-rank statistics. Values of *p* < 0.05 were considered statistically significant. Tumor volume, BED of dose delivered, systemic therapy and primary histology were the variables analyzed for correlation with outcomes. Statistical calculations were conducted using GraphPad Prism (La Jolla, CA) and STATA (StatCorp LP, TX).

## Results

From March 2005 to January 2017, 427 patients with 568 liver metastases treated with SBRT at 25 academic and community-based centers were included in this study. Median age was 67 years (range 31–91 years). Colorectal adenocarcinoma (CRC) was the most common primary tumor type (44%), followed by lung (12%), breast (10%), other gastrointestinal (GI) (7%), gynecologic (6%) and pancreatic (5%) tumors. 73% of patients received prior chemotherapy, 17% had undergone surgery and 17% received no prior therapy. In 381 evaluable lesions, there were 2 lesions in the caudate lobe, 56 were central, 72 lesions in the left lobe, 232 right lobe lesions and 19 were in the dome. Median tumor volume was 40 cm^3^ (1.6–877 cm^3^), median SBRT dose was 45 Gy (12–60 Gy) delivered in a median of 3 fractions [1–5]. The patient and treatment characteristics are outlined in Table 1.

**Table 1:** Patient, tumor and treatment characteristics

Variable	Total	%
Patients	427	
Lesions	581	
Gender		
Male	207	48%
Female	218	51%
Not reported	2	1%
Median age (range) in years	67 (31–91)	
Median KPS (range)	90 (40–100)	
Primary Tumor		
Colorectal	189	44.3%
Lung	52	12.2%
Breast	42	9.8%
Gastrointestinal	33	7.7%
Gynecological	26	5.9%
Pancreas	20	4.9%
Other	65	15.2%
Prior Treatment		
Chemotherapy	314	73.5%
Surgery	73	17.1%
Radiation therapy	8	1.8%
Radiofrequency Ablation	9	2.1%
TACE	3	0.7%
Cryotherapy	1	0.2%
No prior treatment	72	16.9%
Median Lesion Volume (range) in cc	40 (1–2116)	
Median Dose (range) in Gy	45 (12–60)	
Median BED (range) in Gy	100 (16.8–180)	
Median Number of Fractions (range)	3 (1–5)	

**Fig. 1:**
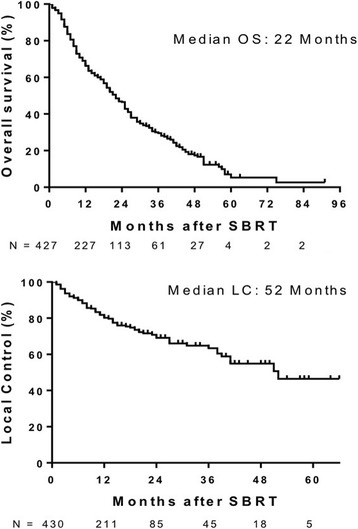
Overall survival (top panel) and local control (bottom panel) of patients with liver metastases treated with SBRT

**Fig. 2:**
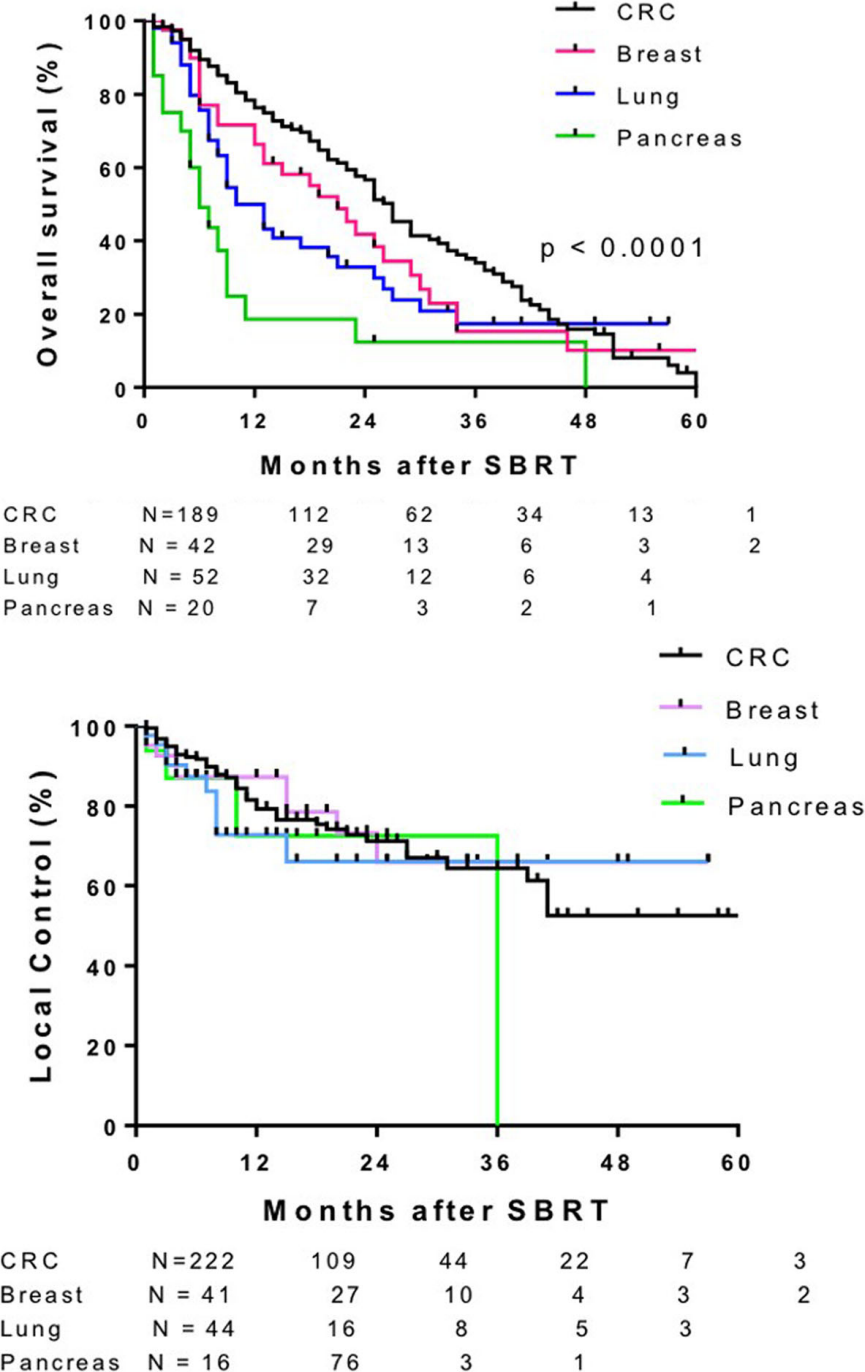
Overall Survival (top panel) and local control (bottom panel) of liver metastases treated with SBRT shown by histology

**Fig. 3:**
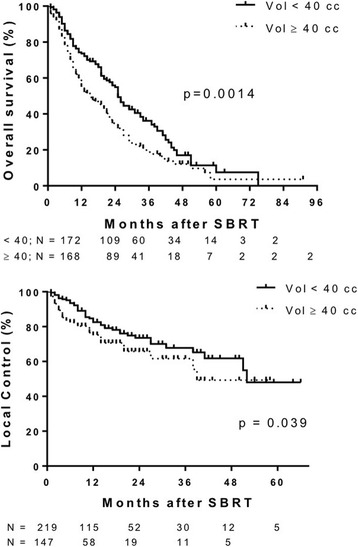
Overall survival (top panel) and local control (bottom panel) of liver metastases treated with SBRT by target volume

**Fig. 4:**
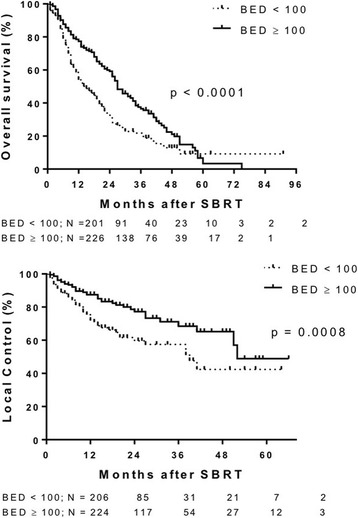
Overall survival (top panel) and local control (bottom panel) of liver metastases by dose

**Fig. 5:**
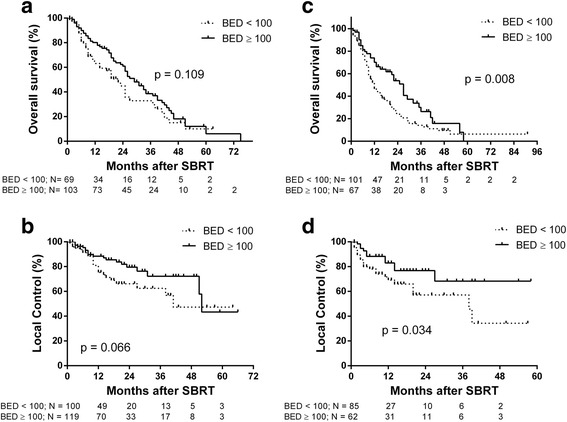
Overall survival and local control of patients with liver metastases volumes < 40 cm^3^ (**a**), (**b**) and ≥ 40 cm^3^ (**c**) and (**d**) by dose (≥ 100 BED vs. < 100 BED). Overall survival and local control are significantly better for tumors ≥ 40 cm^3^ treated with BED ≥ 100 compared to BED < 100 (panels C, (**d**)

Median follow up was 14 months (range 3–91 months). Median OS was 22 months (Fig. 1 Top Panel). Median OS was greater in patients with primary CRC (27 months), breast (21 months) and gynecological (25 months) tumors compared to lung (10 months) and pancreatic (6 months) primary tumor types (*p* < 0.0001). One-year survival for primary CRC, breast, gynecologic, lung, other GI and pancreatic tumor types was 76.4%, 66.4%, 81.3%, 50%, 61% and 18%, respectively (Fig. 2 top panel). Median OS of patients with liver metastases < 40 cm^3^ was 25 months vs 15 months for patients with volumes ≥40 cm^3^ (*p* = 0.0014) (Fig. 3 top panel). Higher BED_10_ was also associated with improved OS with median OS of 27 months for BED_10_ ≥ 100 Gy compared to 15 months for BED_10_ < 100 Gy (*p* < 0.0001) (Fig. 4 Top panel). Unlike tumors < 40cm^3^, LC and OS were also particularly significantly better for tumors >40cm^3^ when the BED was > than 100 (Fig. 5 Panels C&D). The use of systemic therapy did not correlate with survival in this cohort.

LC was evaluated in 430 liver metastases from 324 patients. Median duration of LC was 51 months (Fig. 1 bottom panel). LC did not differ for different primary histology when controlled for volume or dose (Fig. 2 bottom panel). In general, small tumors (< 40 cm^3^) also had improved LC with median LC rates of 52 months and 39 months for tumor volumes < 40 cm^3^ vs ≥ 40 cm^3^, respectively (Fig. 3 bottom panel). Median LC for BED_10_ ≥ 100 Gy was 52 months compared to 39 months for BED_10_ < 100 Gy (*p* < 0.0001). One- and 2-year LC rates for BED_10_ ≥ 100 Gy were 87.5% and 77.2%, respectively, compared to 1- and 2-year LC rates for BED_10_ < 100 Gy of 71.8% and 59.6%, respectively (Fig. 4 bottom panel).

Toxicity data was not available from all centers for all patients due to the nature of this study. All centers reported fatigue in majority of their patients. Most patients with nausea had lesions treated in the left lobe. There were no grade 3 or higher toxicity reported from any on the treating institutions.

## Discussion

This retrospective registry study which included 427 patients with 568 liver metastases demonstrates reasonable median overall survival (22 months) and durable local control (median 52 months). Tumor volumes, primary histology and SBRT dose and fractionation regimens varied across centers. Despite this heterogeneous cohort, we observed OS rates comparable to other published reports of SBRT for liver metastases (Table 2). The diversity in tumor and treatment characteristics in this cohort allows us to determine their significance in this unselected real world multi-institutional setting.

**Table 2:** Outcomes in published leterature for SBRT for Liver metastasis

Study	Number of lesions	Number of patients	Primary	Dose/fractionation	Toxicity	Median follow Up (Months)	Local control	Survival
Blomgren et al. [24]	Variable	31	Mixed	8-66Gy/1–4	2 Hemorrhagic Gastritis	1.5–3.8	80%	NR
Herfarth et al. [25]	1–3	37	NR	14–26 Gy/1	NR	Mean 14.9	18 m:67%	1 yr.:76% 2 yr.:55%
Hoyer et al. [26]	1–6 (< 6 cm)	44	Mixed Majority CRC	45Gy/3	1 Liver Failure 2 severe late GI	52	2 yr.: 86%	1 yr.:67 2 yr.:38
Mendez Romero et al. [27]	1–3 < 7 cm)	25	Mixed Majority CRC	37.5Gy/3	4 acute Grade ≥ 3 1 late Grade 3	12.9	2 yr.: 86%	1 yr.:85% 2 yr.:62%
Rusthoven et al. [29]	1–3 (< 6 cm)	47	Mixed Majority CRC	60Gy/3	< 2% Late Grade ≥ 3	16	2 yr. 92% < 3 cm:100%	Median 17.6
Lee et al. [30]	Variable	68	Mixed Majority CRC	28-60Gy/3	8 acute Grade 3 1 Grade 4	10.8	1 yr.: 71%	18 m:47%
Ambrosino et al. [37]	1–3 (< 6 cm)	27	Mixed Majority CRC	25-60Gy/3	NR	13	74%	NR
Goodman et al. [23]	1–5 (< 5 cm)	26	Mixed Majority CRC	18-30Gy/1	4 late Grade 2	17.3	1 yr.:77%	1 yr.:62% 2 yr.:49%
Rule et al. [31]	1–5	27	Mixed Majority CRC	30Gy/3 50-60Gy/5	No ≥ Grade 2	20	30Gy56% 50Gy:89% 60Gy:100%	30Gy56% 2 yr. 50Gy:67% 2 yr. 60Gy:50% 2 yr
Scorsetti et al. [39]	1–3 (< 6 cm)	61	Mixed Majority CRC	52.5–75/3	No ≥ Grade 3	24	91%	1 yr.: 80% 2 yr.:70%
Present Study	Variable	427	Mixed Majority CRC	45(12–60)/3(1–5	NR	14(1–91)	Median:52 m 1 yr.: 84% 2 yr.:72%	Median:22 m 1 yr.: 74% 2 yr.:49%

One of the first reports of the use of SBRT in extracranial tumors was published over 20 years ago by the investigators from Karolinska Institute in Sweden, who reported on the first 42 lesions of the lung, liver, and retroperitoneal space in 31 patients treated with “stereotactic high-dose fraction radiation therapy”. With a wide range of doses and fractionations, ranging from 1 to 4 fractions, the local control rate was 80%, and 50% of the tumors either decreased in size or disappeared [24]. These data fueled further interest in this SBRT approach, and multiple trials have subsequently evaluated the use of SBRT for liver tumors.

Early studies of liver SBRT investigated single-fraction regimens, extrapolating from the single-fraction approach used in radiosurgery for the brain. Investigators from the University of Heidelberg were the first to report prospective outcomes of single-fraction SBRT for liver metastases [25]. A total of 37 patients, with 55 liver metastases, were treated with single- fraction SBRT on a dose-escalation scheme starting at 14 Gy and increasing to 26 Gy. The 18-month local control rate was 67% for all patients, but it was significantly higher for patients treated at 22–26 Gy vs those treated at 14–20 Gy (81% vs 0%). The investigators did present the caveat that this result may have been due to a learning phase, as investigators had noted that local control also improved in patients who were enrolled later in the study, as more appropriate margin expansions were applied. No significant toxicity was reported. Stanford University Medical Center performed a phase I single-fraction dose-escalation study for primary and metastatic liver tumors [23], which included 19 of 26 patients with hepatic metastases. The single-fraction radiation dose was escalated from 18 to 30 Gy in 4-Gy increments. At a median follow-up of 17 months, there were no dose-limiting toxicities. There was one acute grade 2 (duodenal ulcer 1 month after treatment) and two late grade 2 gastrointestinal toxicities, and both duodenal ulcers manifested by gastro- intestinal bleeding, at 8 months and 25 months post treatment. Of note, 20 months after treatment, the second patient received an additional 56 Gy using intensity-modulated radiotherapy with conventional fractionation to the porta hepatitis region for a recurrence in the treated (18 Gy) lesion and developed an ulcer, 3 months after completion of the additional radiotherapy. All 3 of the duodenal ulcers were among patients treated to sites in the porta hepatis. The 1- year cumulative incidence of local control for all patients was 77%. For patients with liver metastases, the 1-year and 2- year overall survival rates were 62% and 49%, respectively. Although the results of single-fraction liver SBRT appeared promising [32], the potential toxicity of ultra-high-dose radiotherapy in the abdomen led many groups to investigate hypofractionated regimens. Hoyer et al. reported outcomes of 44 hepatic metastases treated with SBRT 45 Gy in 3 fractions, with a 2-year actuarial local control of 79%. One and 2-year overall survival was 67% and 38%, respectively [26]. Treatment-related toxicity included 1 patient who died of hepatic failure, 1 patient with colonic perforation requiring surgical management, and 2 patients with duodenal ulceration treated conservatively. Of note margins to PTV (planning target volumes) were used for respiratory motion and tracking or gating to manage respiratory motion was not used in this study which could account for the high peri-hepatic gastrointestinal toxicity. A phase I/II study of 3-fraction SBRT in patients with primary and metastatic liver lesions was conducted in the Netherlands. A total of 34 liver metastases were treated to 37.5 Gy in 3 fractions with a 2-year local control rate of 86%. One and 2-year overall survival was 85% and 62%, respectively. Our data compare favorably with these series.

A summary of select prospective trials using SBRT for liver metastases is presented in Table 2 and is described in more detail. To date, there are no published phase III data. The studies, as in our current database study, vary in dose heterogeneity, primary histology, tumor volumes, total radiation dose, dose per fraction. However, in the largest series of its kind, in our study, patients were treated with relatively homogenous dosimetric planning criteria with consistent and reliable respiratory motion management [21].

Investigators at the University of Colorado performed a prospective phase I/II trial of 3-fraction SBRT for patients with 3 or fewer liver metastases, measuring less than 6 cm. In the phase I portion of the trial, which included 18 patients, the dose of SBRT was escalated from 36 to 60 Gy in 3 fractions, and no dose- limiting toxicity was observed [28]. In the subsequent report of the combined phase I/II multi-institutional results, 47 patients were treated to 63 liver metastases at 7 participating institutions [29]. 38 patients received the phase II dose of 60 Gy in 3 fractions. With a median follow-up of 16 months, the 1- and 2-year actuarial in-field local control rates were 95% and 92%, respectively. Among lesions < 3 cm, the 2-year actuarial local control was 100%. The 2-year overall survival rate was 30%, and only 1 patient experienced grade 3 or higher toxicity (2%). Another prospective study reported the use of SBRT in 72 patients with 182 treated sites of metastases [37]. Patients had a median of 2 lesions (range: 1–6) with a median diameter of 2.7 cm (0.5–12.2 cm). The median total dose was 45 Gy (17.5–56 Gy) delivered in 2–10 fractions. With a median 12-month follow-up, they reported an 88% in-field local control rate and no grade 3 or higher toxicity. A phase I study of 6-fraction SBRT for liver metastases was performed at Princess Margaret Hospital [30]. The dose was determined by the V_eff_ irradiated and the risk of RILD. Individualized radiation doses were based on normal tissue complication probability (NTCP)-calculated risk of RILD at 3 risk levels (5%, 10%, and 20%). The median SBRT dose was 41.8 Gy in 6 fractions over 2 weeks. Among 68 patients, there were only two grade 3 liver enzyme changes, but no RILD or other grade 3 or higher toxicity. With a median follow-up of 10.8 months, the 1-year local control rate was 71%, and the 18- month overall survival rate was 47% (38)The authors commented in their discussion that the use of V_eff_ might have led to an overestimation of toxicity risk and thus overly conservative prescription doses, and they cautioned against an overreliance on models to convert dose constraints applicable to conventionally fractionated radiotherapy to the SBRT setting. A prospective study from Italy evaluated 27 patients with liver metastases treated with 25–60 Gy (median 36 Gy) delivered in 3 fractions [38]. Mean tumor volume was 35.9 ml. At a median follow-up of 13 months, crude local control was 74%. Mild-to-moderate transient hepatic dysfunction was observed in 9 patients, pleural effusions in 2, and partial portal vein thrombosis, pulmonary embolism, and upper gastrointestinal tract bleed in 1 patient each. The University of Texas Southwestern reported results from their phase I SBRT dose-escalation trial, with 3 dose groups, 30 Gy/3 fractions, 50 Gy/5 fractions, and 60 Gy/5 fractions [31]. At 2 years, local control was 56%, 89%, and 100%. Two-year overall survival was 56%, 67%, and 50% accordingly. Further, there appeared to be a significant dose- response relationship between 30 and 60 Gy (*P* = 0.009). There were no grade 4–5 toxicity and one grade 3 asymptomatic transaminitis occurred in the 50 Gy cohort.

More recently, the group from Milan reported findings from a phase II trial including 61 patients with 76 liver metastases treated to 25 Gy in 3 fractions [39]. At a median follow-up of 12 months, the overall local control rate was 95%. One and 2-year overall survival was 80% and 70%, respectively. There were no reported RILD; 1 patient experienced late grade 3 chest wall pain.

The Radiation Therapy Oncology Group (RTOG) has conducted a phase I trial of hypofractionated RT for hepatic metastases (RTOG 0438) and is currently presented in abstract form only. A total of 26 patients were enrolled, and 4 dose levels were achieved: 35–50 Gy in 5 Gy increments delivered in 10 fractions. There were no dose-limiting toxicities reported. Four patients (2 patients at 45 Gy and 2 patients at 50 Gy) developed grade 3 toxicity.

Several studies have evaluated potential prognostic factors for local control with SBRT for liver metastases. Smaller tumors and those receiving a higher dose have been associated with better local control [25, 29, 30]. This appears to be validated in this large cohort of patients treated with varying dose fractionation schemes, when normalized for BED. While smaller tumors may have been treated with higher doses due to the ability to treat normal liver to tolerance in smaller tumors, they appear to be independently predictive for local control and survival in this study. In larger tumors it was significant in our study thea higher BED delivered translated into better local control and survival. A meta-analysis of prognostic factors following SBRT for colorectal liver metastases demonstrated that total dose of radiation, dose per fraction, and the biologically effective dose were significantly associated with local control [40]. Local control rate exceeding 90% was achieved when doses of 46–52 Gy in 3 fractions were delivered, and the authors concluded that doses of 48 Gy or higher in 3 fractions should be offered if feasible. Interestingly, there have been several studies indicating that histology may affect outcomes with colorectal metastases having worse local control than metastatic lesions from other primary sites; thus, the higher dose of 48 Gy may be necessary in these tumors while lower doses may be sufficient for noncolorectal histologies [25, 41–43]. However, in our study colorectal metastasis did not fare particularly inferiorly. This could have been due to selection bias and other unreported factors including extent and status of systemic disease and varying use of systemic therapy [43].

Another unique feature of this study is that all patients were treated with the CyberKnife® Stereotactic Radiosurgery System with Synchrony® Respiratory Tracking System. The CyberKnife with Synchrony Tracking System uses image-guidance and real-time motion management to accurately deliver high doses of radiation to tumors that move with respiration [21, 44]. A potential advantage of the CyberKnife System using Synchrony is the capability to track tumor motion, leading to a reduced margin (typically 3–5 mm) in comparison with linac-based systems that use abdominal compression, breath-hold techniques or respiratory gating, where the tumor position is generated from different phases of respiration and typically include a 5–10 mm margin to compensate for tumor motion with an addition small margin for set-up uncertainty. This reduction in margin may spare adjacent normal liver from receiving high doses of radiation, potentially resulting in lower toxicities. The decreased volume of liver receiving 15 Gy (V_15_) and reduced mean liver doses can be achieved with Synchrony type real time respiratory motion management, thereby decreasing likelihood of RILD and allowing higher does to be delivered for larger tumors. Its accuracy in respiratory motion management [45, 46] and relative ability to spare normal liver tissue in comparison to other respiratory motion management techniques has been reported and future studies need to be done to investigate the long-term clinical outcomes. In principle SBRT treatments are designed to deliver maximum prescribed dose to the tumor with a rapid fall off of dose to the surrounding normal tissue. A potential advantage of the CyberKnife System is its intrinsic ability to create a treatment plan which includes hundreds of non-isocentric beams cumulating the dose in target volume with a rapid dose fall off. Studies have shown improvements in treatment plan quality when using such non-coplanar beams with sufficient quality and quantity [47]. There is scarcity of literature on the comparative clinical outcomes and toxicity of various SBRT techniques [48]. Furthermore, future studies need to be done to assess the effects of SBRT on long-term outcomes on tumors located near the porta hepatis.

Patient registries, like RSSearch®, have the ability to accumulate data from a large number of patients in a relatively short time and thus, outcomes from patient registries can be reported years before outcomes of prospective clinical trials [36]. Studies from patient registries will not replace randomized clinical trials, however outcomes from patient registries can complement cooperative group studies as well as generate hypotheses for future studies [33, 34].

This observational study is one of the largest patient registries to report treatment management practices of SBRT for liver metastasis in a real-world setting. The demonstration of the ability to achieve outcomes in this unselected cohort in a multiinstitutional setting, comparable to prospective single institution series and protocols, makes these results more generalizable. It adds to the increasing body of evidence for SBRT for liver metastasis and provides impetus for future prospective studies to define the optimal role of SBRT for liver metastasis. However, there are many limitations to this retrospective study including heterogeneity in patient, tumor and treatment characteristics and the potential bias in selection and use of other treatment modalities including systemic therapy. However, the most significant limitation of this study, because of its inherent nature, is the lack of completeness of toxicity data. The available reported data seems to be consistent with the existent literature.

## Conclusions

SBRT provides good OS and LC for metastatic liver lesions. Higher SBRT doses (BED_10_ ≥ 100 Gy) and smaller tumor volumes (< 40 cm^3^) are associated with improved LC and OS. Patients with liver metastases from CRC, breast and gynecological primary tumors tend to have better OS compared to lung and pancreatic primary tumor types, but this could be attributed to selection bias or differences in biology and use of systemic therapy. Future prospective trials assessing the impact of histology and dose with the combination of systemic and immune therapies are needed to help define the role for SBRT to improve outcomes.

## Abbreviations

BED: Biologically Equivalent Dose
GTV, CTV, PTV: Gross, Clinical and Planning Target Volume
Gy: Gray
NTCP: Normal Tissue Complication Probability
OS, LC: Overall Survival, Local Control
RILD: Radiation Induced Liver Damage
RTOG: Radiation Therapy Oncology Group
SBRT: Stereotactic Body Radiotherapy
